# Molecular mechanisms of renal cell carcinoma metastasis and potential targets for therapy

**DOI:** 10.3389/fcell.2025.1521151

**Published:** 2025-01-20

**Authors:** Xinwei Li, Wei Xiong, Zhiyong Xiong, Xiaoping Zhang

**Affiliations:** ^1^ Department of Urology, Union Hospital, Tongji Medical College, Huazhong University of Science and Technology, Wuhan, China; ^2^ Institute of Urology, Union Hospital, Tongji Medical College, Huazhong University of Science and Technology, Wuhan, China; ^3^ Department of Nephrology, Union Hospital, Tongji Medical College, Huazhong University of Science and Technology, Wuhan, China; ^4^ Shenzhen Huazhong University of Science and Technology Research Institute, Shenzhen, China

**Keywords:** renal cell carcinoma, metastasis, metastatic renal cell carcinoma, molecular mechanisms, targeted therapy

## Abstract

Renal cell carcinoma is a common type of cancer, with approximately 30% of patients potentially developing metastatic disease. Some patients with metastatic renal cell carcinoma are found in advanced stages, so the 5-year survival rate for metastatic renal cell carcinoma is only 14%. Currently, there are several drugs available for patients with metastatic renal cell carcinoma, and their overall survival can be extended to nearly 5 years. However, the sensitivity and efficacy of the treatment are still unsatisfactory. New targets and drugs to improve patient prognosis are urgently needed, but these are closely linked to the molecular mechanisms of renal cell carcinoma metastasis. In this review, we present the definition and common molecular mechanisms of metastatic renal cell carcinoma and provide new insights on their potential link to targeted therapies, which may enlighten scientists to develop future targeted therapeutic agents to improve the prognosis of patients with metastatic renal cell carcinoma.

## 1 Introduction

Renal cell carcinoma (RCC) is a common cancer worldwide, with 81,610 new cases and 14,390 deaths expected in the United States in the year 2024 (Siegel et al., 2024). As for metastatic kidney cancer, the median overall survival of metastatic renal cell carcinoma (mRCC) patients currently treated with immune checkpoint inhibitors (ICIs) is estimated to be approximately 50 months (Park et al., 2024). To date, mRCC remains one of the most challenging aspects of renal cancer treatment. Targeted therapy for metastatic tumors, as an emerging approach, holds broader application prospects and is crucial for improving patient outcomes. The foundation of targeted therapy lies in identifying new therapeutic targets. The mechanisms driving RCC metastasis are intricate and involve multiple pathways and molecules, as reported in the literature (Ganesh and Massagué, 2021). At the molecular level, related studies have shown that tumor metastasis depends on clonal selection, the potential of metastatic cells to dynamically transition to different states, and the ability to exploit the immune environment (Gerstberger et al., 2023). Up to now, a few reviews have focused on the molecular mechanisms of RCC progression, but no reviews have focused on the summary of mechanisms of RCC metastasis and linked them to targeted therapy particularly. In this review, we summarized the definition and common molecular mechanisms of renal cell carcinoma metastasis and proposed new insights into its potential connection with targeted therapies, which may enlighten future targeted therapy drug development for improving the outcome of mRCC patients.

## 2 Clinical background of renal cell carcinoma

Clear cell renal carcinoma (ccRCC) accounts for 70%–80% of all pathologic types of RCC, with other types of RCC including papillary, chromophobe, medullary, collecting duct, microphthalmia (MiT) family translocation, succinate dehydrogenase–deficient, hereditary leiomyomatosis and syndrome-associated, and unclassified RCC (Barata et al., 2024; Wang et al., 2024). The most common symptoms of kidney cancer are hematuria or microscopic hematuria, lateral dorsal or flank pain, and palpable abdominal mass. However, due to its retroperitoneal location, RCC can grow significantly without causing symptoms. In some cases, metastatic lesions are detected before the primary kidney tumor is identified through systemic examination and pathology (Petejova and Martinek, 2016; Singla et al., 2022). The initial diagnosis of RCC relies on imaging modalities, and enhanced CT and MRI provide strong evidence for the detection of malignant mass (Bahadoram et al., 2022). Liquid biopsy has made significant progress in RCC through the use of circulating tumor cells (CTCs), ctDNA/cfDNA, cfRNAs, exosomes, and tumor-derived metabolites or proteins, with advancements in next-generation sequencing (NGS), droplet digital PCR (ddPCR), and methylation analysis enhancing the accuracy and applicability of ctDNA/cfDNA. However, challenges remain, including low CTC levels, difficulty distinguishing ctRNAs from cfRNAs, complex miRNA regulatory networks, and insufficient standardization of biomarker analysis (Li M. et al., 2023). Imaging diagnostics for RCC have seen advancements with agents like ^68^Ga-DPI-4452 and ^68^Ga-FAPI-PET/CT showing high sensitivity and specificity for detecting ccRCC, alongside CD8 PET imaging for predicting responses to immune checkpoint inhibitors (ICIs). Nonetheless, the long clearance time of high molecular weight tracers increases patient radiation burden, and small molecule tracers with rapid clearance are in need (Ali et al., 2024). While kidney biopsy is essential for histopathologic confirmation in certain cases, its use is controversial due to concerns about influencing treatment decisions, potential complications like bleeding and infection, and questions about accuracy and safety. Standard treatments like nephrectomy often provide the necessary histopathologic evidence (Bahadoram et al., 2022). Of all patients diagnosed with RCC, 20%–30% have metastasis at initial presentation, yet 20%–40% of patients with RCC still develop recurrence and metastasis after radical nephrectomy (Teishima et al., 2023; Vig et al., 2020).

For non-metastatic renal mass, the preferred treatment is surgical resection. For specific patients, a kidney-preserving partial nephrectomy prioritizing the achievement of negative surgical margins is recommended. Based on clinical indications, radical nephrectomy is indicated for patients with an increased oncologic risk as well as for patients scheduled for targeted therapy (Gray and Harris, 2019). In recent years, several agents have been developed to improve the prognosis of metastatic and recurrent RCC patients. These agents target the vascular endothelial growth factor (VEGF) such as bevacizumab and its receptor (VEGFR) such as sunitinib, mammalian target of rapamycin (mTOR) such as temsirolimus, immunocheckpoints programmed death-1 (PD-1) such as nivolumab and its ligand (PD-L1) such as atezolizumab, and cytotoxic T lymphocyte antigen 4 (CTLA-4) such as ipilimumab (Aurilio et al., 2019). Although advances have been made in some aspects of the diagnosis, screening, and treatment of RCC, the survival of patients with metastatic RCC remains unsatisfactory compared to other cancers (Ryalen et al., 2023; Wu et al., 2023a). In addition, drug resistance and side effects of drug therapy are shortcomings that cannot be ignored (Li X. et al., 2024). This means that more researches are still in need to discover new targets, optimize existing therapies, and ultimately improve the outcome and quality of life of mRCC patients.

## 3 Pathology of metastatic renal cell carcinoma

Hematogenous metastasis is one of the most common modes of metastasis for kidney cancer, and other common modes of RCC metastasis include lymphatic metastasis and direct extension (Huang et al., 2017). The most common sites of metastasis for renal cell carcinoma are the renal vein and its branches, the inferior vena cava, lung, bone, liver, lymph nodes, adrenal gland, and brain (Liu et al., 2021; Matuszczak et al., 2023). In recent years, metastasis of renal cell carcinoma to the larynx, mandible, eyelids, small intestine, appendix, and penis has also been reported in the literature (Ahmed et al., 2023; Bellouki et al., 2023; Cho et al., 2024; Elmusa et al., 2022; Huang and Yu, 2023; Ushakova and Ravakhah, 2023). The TNM staging system of renal cell carcinoma is considered to be the criterion for evaluating the metastasis of RCC, which contains tumor (T), node (N), and metastasis (M) classification. Localized RCC (LRCC) stands for RCC staging T_1-2_N_0_M_0_, advanced RCC consists of the remaining stages, while metastatic renal cell carcinoma is defined as tumor invasion through the perirenal fascia and/or presence of distant metastasis (Chen et al., 2021; Zhang et al., 2022). An interesting study showed that histological subtype, nuclear grade, and sarcomatoid differentiation are important predictors of metastasis in patients with RCC (Park, 2023). Besides, another retrospective cohort study showed that RCC WHO/ISUP grades are associated with metastasis, with the high-grade group (Grade 3–4) being more likely to metastasize than the low-grade group (Grade 1–2) (Fujita et al., 2022).

## 4 Molecular mechanisms of renal cell carcinoma metastasis

### 4.1 Signaling pathway activation and inhibition

#### 4.1.1 Wnt/β-catenin signaling pathway

The overexpression of centromere protein A (CENPA) would regulate the cell cycle and activate the Wnt/β-catenin signaling pathway, resulting in ccRCC proliferation and metastasis (Wang et al., 2021). Moreover, Sun et al. found the Follistatin-like 3 (FSTL3) regulates the GSK-3β/β-catenin and BMP1/SMAD pathways, enabling RCC proliferation and metastasis (Sun et al., 2021). In addition, a lncRNA named SLERCC inhibits RCC progression and metastasis by inhibiting the Wnt/β-catenin signaling pathway and directly binding to UPF1. However, high DNMT3A expression recruits DNMT3A to the SLERCC promoter region, inducing aberrant hypermethylation and ultimately inhibiting SLERCC expression (Mao et al., 2022).

#### 4.1.2 PI3K/AKT signaling pathway

Mao and colleagues reported that ciRS-7 can act as a sponge for miR-139-3p, a microRNA that inhibits RCC cell proliferation, migration, and invasion. However, based on targeting TAGLN, ciRS-7 can activate the PI3K/AKT signaling pathway by regulating the miR-139-3p/TAGLN axis, thus assisting the proliferation and metastasis of RCC cells (Mao et al., 2021). Additionally, DEP domain containing 1 (DEPDC1) promotes glycolysis in RCC via the AKT/mTOR/HIF1α pathway, which in turn affects tumor metastasis and TKI resistance (Di et al., 2024). Another study found that tescalcin promotes cell proliferation, migration, and invasion via the NHE1/pHi axis and AKT/NF-κB signaling pathway (Luo et al., 2019). Besides, AGK also promotes RCC metastasis through the PI3K/AKT pathway as mentioned above (Zhu et al., 2020). Moreover, centrosomal protein 55 (CEP55) could promote the upregulation of E-cadherin and downregulation of N-cadherin and ZEB1 via PI3K/AKT/mTOR pathway, resulting in RCC epithelial-mesenchymal transition (EMT), proliferation, and metastasis (Chen et al., 2019).

#### 4.1.3 NF-κB signaling pathway

Researchers found that tumor necrosis factor α (TNFα) activates the NF-κB pathway in RCC cells, leading to p65 binding at the Rictor promoter and ultimately promoting cancer metastasis (Sun et al., 2016). Further, metastasis-associated gene 1 (MTA1) is overexpressed in RCC and regulates the expression of MMP2/MMP9 as well as E-calmodulin through the NF-κB signaling pathway, leading to RCC migration and invasion (Lv et al., 2020). Moreover, sphingosine 1-phosphate (S1P) promotes the proliferation, migration, and EMT of RCC cells through activation of its receptor S1PR3, thereby accelerating RCC carcinogenesis and metastasis. This process involves the S1PR3/Gi/p38/Akt/p65/cyclin D1-CDK4 signaling pathway to regulate cell proliferation, and the S1PR3/Gi/q/ERK/p38/p65 signaling pathway to regulate cell migration (Yan et al., 2022). As mentioned, Tescalcin is also associated with NF-κB signaling pathway (Luo et al., 2019).

#### 4.1.4 Other pathways

Metadherin (MTDH) activates SND1 to mediate ERK signaling and EMT, thereby promoting migration and metastasis (He et al., 2020). Additionally, MRCCAT1, a key lncRNA, inhibits NPR3 transcription by recruiting PRC2 to the NPR3 promoter region, therefore activating the p38-MAPK signaling pathway and promoting ccRCC metastasis (Li et al., 2017). Furthermore, ApoC1 could promote the activation of STAT3 and enhance the metastasis of ccRCC. Meanwhile, exosomes could transfer ApoC1 from the ccRCC cells to the vascular endothelial cells to promote tumor angiogenesis and metastasis via STAT3 pathway (Li et al., 2020).

### 4.2 Gene expression regulation

#### 4.2.1 Non-coding RNA regulation

Scientists have found that miR-148a-3p targets circUBAP2 in ccRCC, with its expression level negatively correlating with that of circUBAP2. The miR-148a-3p could reverse the inhibitory effect of circUBAP2 on ccRCC cell proliferation, migration, and invasion, and it could also target FOXK2 to affect ccRCC proliferation and metastasis (Sun et al., 2020). Dong’s team has found that lncRNA ZFAS1 could promote ccRCC growth and metastasis through the miR-10a/SKA1 pathway (Dong et al., 2019). Similarly, miR-100 promotes autophagy and inhibits migration and invasion of RCC cells by targeting NOX4 and inhibiting the mTOR signaling pathway (Liu et al., 2022). Besides, miR-139-3p would be inhibited by ciRS-7 to promote RCC metastasis (Mao et al., 2021).

Regarding lncRNAs, lncHILAR upregulates Jagged-1 and CXCR4 expression by acting as a ceRNA for miR-613/206/1-1-3p, thus activating the Jagged-1/Notch/CXCR4 signaling pathway and promoting RCC cell invasion and metastasis (Hu et al., 2021). Additionally, via binding to the promoter area of ERβ, lncRNA-SERB could regulate ERβ functions through transcriptional regulation of zinc finger E-box binding homeobox 1 (ZEB1), thus promoting vasculogenic mimicry (VM) formation (Tang et al., 2024).

In addition, circSDHC could protect CDKN3 from miR-127-3p inhibition by competitively binding to miR-127-3p, which in turn activated the E2F1 pathway and promoted RCC proliferation and invasion (Cen et al., 2021).

#### 4.2.2 Transcription factor regulation

Lu et al. reported that KLF2 deficiency impairs the transcriptional repression of GPX4, inhibiting ferroptosis and thereby promoting ccRCC cell migration and invasion (Lu et al., 2021). Also, c-Myb could transcriptionally activate miR-520h, which would target MAGI1. Then, MAGI1 could stabilize the PTEN/MAGI1/β-catenin complex to modulate β-catenin signaling pathway, mediating RCC metastasis (Wang W. et al., 2019). Besides, HIF-2 transcriptionally targeted the hypoxia response element on the Polo-like kinase 1 (Plk1) promoter, which promoted Plk1 expression in ccRCC, leading to ccRCC growth, metastasis, and drug resistance (Dufies et al., 2021). Moreover, YBX1 could promote SPP1 expression by interacting with G3BP1, which in turn activates the NF-κB signaling pathway, ultimately leading to increased invasion and metastasis of RCC (Wang Y. et al., 2019). Interestingly, N-acetyltransferase 10 (NAT10) promotes ankyrin repeat and zinc finger peptidyl tRNA hydrolase 1 (ANKZF1) expression through N4-acetylcytidine (ac4C) modification, which in turn regulates YAP1 activity and activates the expression of pro-lymphangiogenic factors to promote lymphangiogenesis and tumor progression in ccRCC (Miao et al., 2024).

### 4.3 Protein modification

Xu et al. found that circPOLR2A forms a UBE3C/circPOLR2A/PEBP1 protein-RNA ternary complex with UBE3C and PEBP1 proteins, which enhances UBE3C-mediated ubiquitination and degradation of PEBP1 proteins, which activates the ERK via ERK1/2 phosphorylation signaling pathway, thereby promoting angiogenesis (Xu et al., 2022). Acylglycerol kinase (AGK) activates the GSK3β S9 phosphorylation site via the PI3K/AKT pathway, leading to GSK3β inactivation, β-catenin stabilization, and subsequent promotion of RCC growth and metastasis (Zhu et al., 2020).

## 5 Mechanisms inhibiting renal cell carcinoma metastasis

### 5.1 Signaling pathway regulation

EF-hand domain family member D1 (EFHD1) binds to MCU through its N-terminal domain, suppressing mitochondrial Ca^2+^ uptake and thereby inactivating the Hippo/YAP signaling pathway (Meng et al., 2023). Similarly, leukemia inhibitory factor receptor (LIFR) attenuates ccRCC metastasis by upregulating Hippo signaling pathway kinase activity, which inhibits YAP expression (Lei et al., 2018). Yin’s team found that HOOK1 could inhibit RCC proliferation, metastasis, angiogenesis, and sunitinib resistance via TNFSF13B/VEGF‐A signaling. Moreover, meletin, an agonist of HOOK1, demonstrates greater antitumor efficacy when combined with sunitinib or nivolumab compared to its use as a monotherapy (Yin et al., 2023). Additionally, MUC15 inhibits RCC cell invasion and metastasis through PI3K/AKT signaling (Yue et al., 2020). Nuclear receptor coactivator 7 (NCOA7) inhibits the MAPK/ERK pathway, regulating EMT and apoptosis and thereby inhibiting ccRCC progression and metastasis (Guo et al., 2023). Similarly, SH3BGRL2 inhibits ccRCC proliferation and metastasis by activating the LATS1/2-YAP-TEAD1 signaling pathway, and TEAD1 promotes EMT through TWIST1 upregulation (Yin et al., 2020). Zhang et al. found that thymoquinone induces autophagy in RCC cells by activating the AMPK/mTOR signaling pathway, which inhibits the EMT and metastasis of RCC cells (Zhang et al., 2018). Likewise, FOXC1 activates the AMPK signaling pathway and inhibits the mTOR signaling pathway by upregulating the expression of ABHD5 to inhibit the growth and metastasis of RCC cells (Li J. et al., 2024).

### 5.2 Transcription factor regulation

The cRAPGEF5 acts as a sponge of oncogenic miR-27a-3p, which targets the suppressor gene TXNIP, thus inhibiting RCC progression and metastasis (Chen et al., 2020). Additionally, melatonin reduces the DNA-binding activity of p65 and p52, thereby inhibiting MMP-9 transcriptionally and affecting its transcriptional activation and cell migration via Akt-mediated JNK1/2 and ERK1/2 signaling pathways. Besides, high MMP-9 expression correlates with a poorer RCC prognosis (Lin et al., 2016).

### 5.3 Protein modification

Ubiquitin-specific peptidase 53 (USP53) prevents the inactivation of the NF-κB pathway by reducing ubiquitination of IκBα, thereby further inhibiting ccRCC proliferation and metastasis (Gui et al., 2021). Likewise, ubiquitin-specific peptidase 2 (USP2) downregulates the NF-κB pathway, inhibiting EMT in clear cell renal cell carcinoma metastasis (Duan et al., 2022). Luo and colleagues discovered that the outer mitochondrial membrane (OMM) protein MFN2 inhibits ccRCC tumor growth and metastasis by binding to the small GTPase Rab21, facilitating interaction with endocytosed EGFR in ccRCC. This process promotes docking of endocytosed EGFR to mitochondria, where it is subsequently dephosphorylated by OMM-resident tyrosine-protein phosphatase receptor type J (PTPRJ), leading to inactivation of the EGFR signaling pathway and attenuation of EGFR oncogenic signaling (Luo et al., 2023). Besides, EFHD1 would also upregulate STARD13 to enhance YAP protein phosphorylation at Ser-127 to suppress cell migration and metastasis (Meng et al., 2023). Moreover, IL6 mediates crosstalk between normal fibroblasts and RCC cells, promoting cell migration via the STAT3 pathway. Conversely, GATA3 reduces STAT3 phosphorylation, inhibiting RCC cell migration (Shi et al., 2020) (Table 1).

**TABLE 1:** Summary of the key mechanisms in promoting and inhibiting RCC metastasis.

	Specific mechanisms	Molecules and pathways involved	Effects
Mechanisms promoting RCC metastasis	Wnt/β-catenin Pathway	CENPA, FSTL3, SLERCC, DNMT3A	Regulates cell cycle, promotes or inhibits RCC proliferation and metastasis
	PI3K/AKT Pathway	ciRS-7, DEPDC1, Tescalcin, AGK, CEP55	Influences RCC cell proliferation, migration, invasion, and TKI resistance
	NF-κB Pathway	TNFα, MTA1, S1P, Tescalcin	Promotes RCC cell proliferation, migration, invasion, and EMT
	Other Pathways	MTDH, MRCCAT1, ApoC1	Activates ERK and p38-MAPK pathways, and promotes RCC migration and metastasis
	Non-coding RNA regulation	miR-148a-3p, lncRNA ZFAS1, miR-100, lncHILAR, lncRNA-SERB, circSDHC	Affects RCC cell proliferation, migration, invasion, and autophagy
	Transcription factor regulation	KLF2, c-Myb, HIF-2, YBX1, NAT10	Modulates target gene expression, influencing RCC cell migration, invasion, and drug resistance
	Protein modification	UBE3C/circPOLR2A/PEBP1 complex, AGK	Affects protein stability, activates specific pathways like ERK and β-catenin
Mechanisms inhibiting RCC metastasis	Pathway regulation	EFHD1, LIFR, HOOK1, MUC15, NCOA7, SH3BGRL2, thymoquinone, FOXC1	Inhibits Hippo/YAP, PI3K/AKT, MAPK/ERK pathways, and reduces RCC migration and invasion
	Transcriptional regulation	cRAPGEF5, melatonin	Inhibits MMP-9 expression, and reduces RCC progression and metastasis
	Protein modification	USP53, USP2, MFN2, EFHD1, GATA3	Reduces NF-κB pathway activity, inhibits EGFR signaling, and weakens RCC growth and metastasis

## 6 Therapeutic approaches and current gaps

Current major targeted therapies and potential drugs under investigation primarily focus on specific signaling pathways, including tyrosine kinase inhibitors (TKIs) and mTOR inhibitors. Pazopanib, a tyrosine kinase inhibitor, enhances the function of dendritic cells (DCs) by inhibiting the Erk/β-catenin pathway. This inhibition leads to the upregulation of maturation markers such as HLA-DR, CD40, and CCR7, reduces IL-10 production and endocytosis, and increases T-cell proliferation. Additionally, pazopanib downregulates PD-L1 expression. These effects collectively improve the antigen-presenting capability and immune-stimulating capacity of DCs, potentially augmenting the immune response in mRCC patients. Furthermore, pazopanib increases the population of circulating CD4^+^ T cells expressing CD137 (4-1BB), suggesting a potentially exploitable immunomodulatory effect that could be leveraged to improve responses when combined with immune checkpoint inhibitors (ICIs) in tailored treatment protocols (Zizzari et al., 2018). Besides, mTOR inhibitors have been the first-line treatment for mRCC. However, since the mTOR signaling axis is activated in only a subset of RCC patients, clinical trials involving mTOR inhibitors are ongoing (Zheng et al., 2021). A recent clinical trial revealed that the on-treatment tumor growth rate was 1.7 times higher for apitolisib (a dual PI3K/mTORC1/2 inhibitor) compared to everolimus (an mTORC1 inhibitor). The estimated half-life for the loss of treatment effect was 16.1 weeks for everolimus and 7.72 weeks for apitolisib, indicating a faster tumor regrowth rate for patients treated with apitolisib, possibly due to rapid resistance development (Moein et al., 2024). Another study found that MC-4, a novel Akt/PKM2 inhibitor, can potentially overcome the limitations of existing mTOR inhibitors. Therefore, combining MC-4 with current treatments may represent a promising new strategy for treating patients with rapidly progressing advanced RCC (Son et al., 2018). An intriguing study found that PI3K-mutant patient-derived xenograft (PDX) models exhibit significant resistance to TKI treatment. However, these PDX models seem to be sensitive to mTOR inhibitor treatment. Thus, combination therapies that incorporate drugs with different mechanisms of action, such as pairing mTOR inhibitors with TKI, could offer greater benefits to patients. Combination therapy strategies simultaneously target multiple signaling pathways and address potential resistance issues arising from monotherapy, thereby enhancing overall treatment effectiveness (Wu et al., 2023b). Currently, no therapeutic approaches specifically target NF-κB signaling in mRCC. However, inhibitors and monoclonal antibodies that target this pathway have been studied in other diseases and may represent a promising therapeutic approach for mRCC (Guo et al., 2024).

However, drug insensitivity is also tricky from molecular apects. A study found that the O-GlcNAcylation of RIPK1 at Ser331, Ser440, and Ser669 regulates its ubiquitination, thereby reducing the formation of the RIPK1/FADD/Caspase-8 complex. This alteration promotes NF-κB activation, ultimately inhibiting sunitinib-induced RIPK-dependent apoptosis in RCC (Zeng et al., 2024). Conversely, another intriguing study found that SEC14L3 not only emerges as a potential therapeutic target but also uncovers an SEC14L3/RPS3/NF-κB positive feedback loop that can inhibit ccRCC progression and sunitinib resistance. Modulating SEC14L3 expression to activate this feedback loop could offer new therapeutic strategies for ccRCC treatment (Jiang et al., 2024). Besides, ZZDHHC2 mediates AGK’s S-palmitoylation, promoting its translocation to the plasma membrane and activation of the PI3K-AKT-mTOR pathway in ccRCC, thereby modulating sunitinib sensitivity. This indicates that targeting ZDHHC2 could enhance the antitumor efficacy of sunitinib in ccRCC (Sun et al., 2023). Moreover, circPTEN suppresses ccRCC progression and resistance to mTOR inhibitors by enhancing PTEN expression through reduced methylation of the PTEN promoter and decreasing GLUT1 expression by lowering its m6A methylation (Zhan et al., 2023). Overall, these molecular mechanisms provide promising therapeutic avenues for enhancing sunitinib sensitivity and overcoming resistance in RCC, although they also carry the risk of drug insensitivity. Rational utilization of these mechanisms and molecules will contribute to the clinical translation of these fundamental discoveries.

## 7 Discussion and future perspectives

While the overall 5-year survival rate for renal cell carcinoma is approximately 76%, this rate plummets to 14% for patients with mRCC (Aldin et al., 2023). To date, over 800 clinical trials on advanced RCC have been registered on https://clinicaltrials.gov/, with the majority focusing on single-agent or multi-agent targeted therapies and immunotherapies. Nevertheless, while drug treatments—primarily targeted therapies and immunotherapies, administered as single agents or in combination—can extend patient overall survival (OS) in advanced kidney cancer, significant improvements in treatment efficacy remain necessary (Semenescu et al., 2023).

**FIGURE 1:**
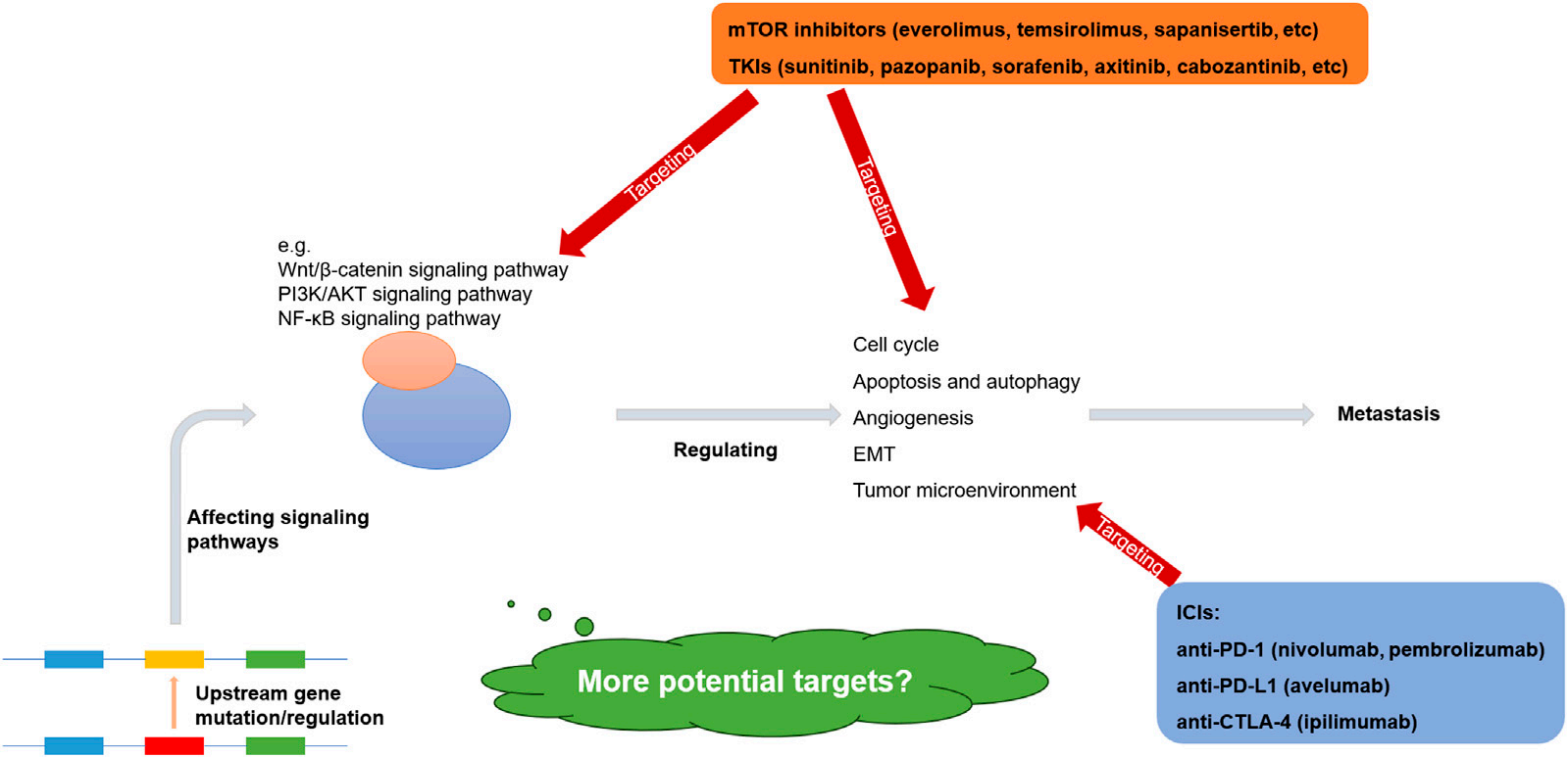
Molecular mechanisms and therapeutic targets of metastatic renal cell carcinoma (Wu et al., 2023b; Sharma et al., 2021; Motzer et al., 2021; Choueiri et al., 2023; Grimm et al., 2024).

The molecular mechanisms underlying renal cell carcinoma metastasis are complex, diverse, and interconnected, with various pathways intersecting. Mutations in upstream genes associated with metastasis are relatively rare and exhibit high variability. In addition, the regulation of non-coding RNAs, the regulation of transcription factors, and protein modifications can be drivers of metastasis as well. However, certain downstream pathways, including the Wnt/β-catenin, PI3K/AKT, and NF-κB signaling pathways, share common features. These pathways regulate the cell cycle, apoptosis, autophagy, angiogenesis, and EMT, contributing to rapid tumor proliferation and metastasis (Ma et al., 2023; Mabeta and Steenkamp, 2022; Mirzaei et al., 2022; Wang, 2021) (Figure 1).

However, current research into the metastatic mechanisms of renal cancer predominantly focuses on RCC cells, with limited studies addressing the tumor microenvironment (TME) where these cells reside. Non-immune cells in the extracellular matrix are inextricably linked to the invasion and migration of cancer cells (Oxburgh, 2022). Immune cells within the TME differentiate and undergo functional changes as tumors progress, suppressing or depleting local tumor immunity and thereby fostering an environment conducive to renal cancer cell metastasis (Mei et al., 2024). A notable study reported that tumor-associated macrophages (TAMs) from VHL-deficient tumors displayed increased *in vivo* glucose uptake, enhanced phagocytic activity, and heightened inflammatory gene expression. Conversely, lymphocytes from Vhl-KO tumors exhibited decreased activation and reduced responsiveness to anti-programmed cell death 1 (anti-PD-1) therapy *in vivo* (Wolf et al., 2024).

Newly identified therapeutic targets offer hope for treating mRCC. Single-nucleus RNA sequencing revealed ceruloplasmin (CP) and proprotein convertase subtilisin/kexin type 6 (PCSK6) as promising ccRCC markers with potential diagnostic and prognostic significance (Wu et al., 2023c). Another study utilized a large sample size and multi-omics techniques to identify UCHL1 expression as one of the potential biomarkers for high-grade tumors with BAP1 mutations, genomic instability, or increased tumor hypermethylation, potentially influencing clinical and therapeutic management (Li Y. et al., 2023).

Future treatment modalities for metastatic renal cell carcinoma hold significant promise. Multi-drug combination therapies have already demonstrated improved patient outcomes compared to monotherapy, suggesting that integrating multiple targeted drugs or combining them with immunotherapy could be a promising direction for future development (Semenescu et al., 2023). Furthermore, genomics and proteomics analyses can predict patient sensitivity or resistance to specific drugs, while the presence or absence of certain biomarkers can guide personalized treatment choices, enabling more precise and individualized care plans (Elias et al., 2021). Moreover, continued in-depth investigation into the shared downstream pathways among different signaling pathways, regulatory mechanisms of gene expression, and cellular interactions within the TME of metastatic renal cell carcinoma could uncover additional therapeutic targets. Additionally, factors within the TME—such as inflammatory responses, hypoxic conditions, metabolic reprogramming, and mechanical stresses—further promote tumor metastasis. These interactions and environmental pressures contribute to genetic and epigenetic heterogeneity, highlighting the need for therapeutic targets that address these components. Importantly, given the relatively poor prognosis of metastatic renal cell carcinoma, developing reliable biomarkers to predict therapeutic response and monitor disease progression is crucial for guiding clinical decision-making. For example, co-deletions involving VHL alongside one or more of the three genes—PBRM1, BAP1, and SETD2—encoding proteins involved in chromatin modification and remodeling, are common and serve as significant co-drivers of tumorigenesis (Walton et al., 2023).

## 8 Conclusion

In conclusion, although significant progress has been made in targeted therapies for metastatic renal cell carcinoma, many challenges still exist. Future studies will require a deeper understanding of the biological process of this disease, which will contribute to the development of more effective treatments to improve patient survival and quality of life.
